# Identification of genes located at breakpoints of uncharacterized chromosomal translocations by the use of chromosomal microdissection and next generation DNA sequencing

**DOI:** 10.1186/1753-6561-7-S2-P55

**Published:** 2013-04-04

**Authors:** Paulo V Campregher, Daniela Borri, Patrícia Severino, Natália Torres, Elvira RP Velloso, Nelson Hamerschlak, Fernando F Costa

**Affiliations:** 1 Albert Einstein Hospital Research Institute, São Paulo, Brazil; 2 Faculdade de Ciências Médicas - Universidade Estadual de Campinas, Campinas, Brazil

## Background

Cancer results from accumulation of pathogenic genetic changes in a somatic cell. Chromosomal translocations (CT) are among the most common molecular changes associated with the development of cancer. The mechanism by which CT causes cancer is through the juxtaposition of two genes, generating a hybrid protein with oncogenic function or through the placing of a proto-oncogene under the control of a promoter region active in the cell of origin. While most genes located at the breakpoints of common CT have been identified, several, less frequent, CT remain uncharacterized.

## Materials and methods

We propose to study a cohort of 17 already identified patients with hematologic malignancies that have uncharacterized CT in the neoplastic cells. To accomplish that, we will perform CT microdissection followed by amplification of chromosomal DNA, and DNA sequencing using next generation DNA sequencing.

## Results

As a pilot study, we have performed microdissection and amplification of a normal chromosome 7, and tested the efficacy of the entire process by doing a PCR for the BRAF gene, which is located on chromosome 7. PCR amplification yielded a specific band with the right molecular weight, attesting the success of our method. In addition, we have successfully microdissected uncharacterized CT of 8 patients, and successfully amplified the DNA of two of these cases.

## Conclusion

We are developing a platform for rapid and robust identification of genes present at breakpoints of uncharacterized CTs, and will identify genes involved in CT of 17 patients with hematological malignancies.

## Financial support

Hospital Albert Einstein.

